# The Efficacy and Feasibility of an Interoceptive Exposure Technique for Preventing the Transition From Subacute to Chronic Back Pain by Altering the Emotional Response to Pain: Protocol for a Pilot Randomized Controlled Trial

**DOI:** 10.2196/45701

**Published:** 2023-10-19

**Authors:** Tage Ingemar Orenius, Leena Ristolainen, Esko Silén, Heikki Hurri

**Affiliations:** 1 Orton Orthopaedic Hospital Helsinki Finland; 2 Research Institute Orton Helsinki Finland

**Keywords:** biomarkers, functional connectivity, interoceptive exposure, low back pain, prevention, subacute

## Abstract

**Background:**

Psychological factors such as stress, anxiety, mood, emotions, cognitive functioning, and pain behavior are relevant to the onset of pain and its continuation in the transition to chronic conditions. Subacute low back pain (LBP), a precursor to chronic LBP, is particularly poorly understood, and its relationships with psychological factors are understudied.

**Objective:**

We will conduct a study aiming to prevent the chronicity of subacute LBP by altering the emotional response to pain using an interoceptive exposure technique (IET). Considering the recent increase in knowledge about psychological risk factors, as well as recent findings in cognitive neuroscience regarding emotional and cognitive background factors in the LBP chronicity process, efforts should be made to discover effective preventive methods.

**Methods:**

We present a novel method aiming to prevent the transition from subacute to chronic back pain by altering the emotional response to pain using an IET. In this pilot randomized controlled trial, participants who are at a higher risk of LBP chronification due to the presence of a biomarker (ie, functional connectivity between the medial prefrontal cortex and nucleus accumbens) will be randomly assigned to the IET intervention group or control group A (treatment as usual). Participants who do not present with the biomarker will be assigned to control group B (treatment as usual) to assess how well this biomarker predicts the chronification of subacute LBP in this study population. Questionnaires measuring the pain experience and psychological distress will be used before the intervention, after the intervention (at 3 months), and at the 12-month follow-up.

**Results:**

This research project will combine 2 novel methods: a biomarker as the inclusion criterion and an IET as the intervention. The comparative study design enables evaluation of the efficacy of the IET, as well as replication of the biomarker’s validity as a possible clinical screening method.

**Conclusions:**

If found to be useful, the IET would offer a cost-effective and reasonable way to develop treatment for acute and subacute back pain conditions. Potential benefits include increased pain management and quality of life for the individual patient with the addition of a potentially useful functional pain management method at the societal level.

**Trial Registration:**

ClinicalTrials.gov NCT05450263; https://clinicaltrials.gov/ct2/show/NCT05450263

**International Registered Report Identifier (IRRID):**

PRR1-10.2196/45701

## Introduction

### Overview

As low back pain (LBP) has been shown to be highly prevalent and hence a major problem throughout the world, preventing LBP from becoming chronic can be considered a major challenge in pain medicine [1]. LBP prevalence rates between 3.9% and 20.3% have been reported [2], with the highest prevalence among female individuals and those aged between 40 and 80 years [3].

Although LBP episodes and associated disability usually improve within weeks, pain and disability typically continue, and recurrences are common [4]. The predictive power of biomarkers for chronic LBP has so far proven to be insufficient [5]. Risk factors for LBP may be genetic, acquired, or due to an individual’s interaction with the environment [6]. In a review of prospective studies, Linton [7] concluded that psychological variables such as stress, distress, anxiety, mood, emotions, cognitive functioning, and pain behavior were relevant to the onset of pain and its continuation in the transition to chronic conditions. The transition from acute localized LBP into chronic widespread LBP has shown relationships with the progression of both peripheral and central sensitization [8]. Furthermore, patients with chronic LBP might develop maladaptive behavioral strategies [9].

Recent brain imaging studies have further supported the role of emotional factors in the LBP chronification process, revealing mental factors influencing pain conditions [10]. Even in acute pain states, emotional factors seem to directly influence neural pain processing, as shown in the brain imaging study by Orenius et al [11], in which a valence-independent emotion interacted with the pain processing in the secondary somatosensory cortex. Functional and emotional nonadjustment factors have been identified in the acute LBP chronification process [12], with emotional nonadjustment relating to helplessness and hopelessness. Furthermore, chronification does not simply implicate the presence of enduring pain symptoms; it signifies a complex process associated with alterations in cortical structure and function. Vachon-Presseau et al [13] emphasized the importance of the emotional brain (ie, the cortico-limbic system) in the modulation of acute pain and in the prediction and amplification of chronic pain. A functional magnetic resonance imaging (fMRI) study showed that chronification of LBP shifts brain representation from nociceptive to emotional neural circuits [14]. These findings indicate the pivotal role that the neural structures involved in emotional learning play in the chronification of back pain [15]. Furthermore, the strength of synchronicity (ie, functional connectivity) between the medial prefrontal cortex (mPFC) and nucleus accumbens (NAc) was predictive (with >80% accuracy) of individuals who would subsequently transition from subacute pain to chronicity at a 1-year follow-up [15].

Neuroimaging studies have shown that cognitive behavioral therapy can change anxiety-related dysfunctions (phobia, traumatic stress, obsessive-compulsive disorder, and panic) of the nervous system [16]. Brain regions associated with attention, interoception, and sensory processing, including the prefrontal cortex and right anterior insula, are thicker in meditation participants than in matched controls, with the thickness of 2 regions correlating with meditation experience [17]. It has also been shown that mindfulness-based stress reduction techniques (a subcategory of cognitive behavioral therapy) produce macroscopic cortical plasticity in the adult human brain, demonstrating a dynamic relationship between behavioral state and cerebral anatomy [18]. Stress reduction correlated with structural changes in the amygdala after an 8-week mindfulness-based stress reduction intervention, demonstrating an association between neuroplastic changes and corresponding improvements in a psychological state variable [19]. An anatomical likelihood estimation meta-analysis showed that 8 brain regions were consistently altered in meditators, including areas key to meta-awareness, exteroceptive and interoceptive body awareness, memory consolidation and reconsolidation, self-regulation and emotion regulation, and intra- and interhemispheric communication [20]. Therefore, psychological interventions can produce both objective structural changes and alterations in the neural processing of emotions.

The treatment of chronic LBP has not primarily focused on removing an underlying organic disease but rather, within a biopsychosocial framework, on identifying the underlying mechanisms driving the disorder and thus enabling treatment to favorably influence the outcome [21]. Psychological methods targeting therapeutic processes relate to attention, cognition, emotions (including emotion regulation), and overt pain behaviors [22]. Despite a growing knowledge of relevant background factors in the transition from acute to chronic LBP [23], insight into preventive psychological methods applicable to subacute (4-12 weeks) LBP, a precursor to chronic LBP, is still particularly scarce. Insufficient theoretical guidance and integration in the design, selection, and delivery of methods that precisely target known dysfunctional behavioral processes have been presented as plausible shortcomings [24]. A recent systematic review showed that psychological interventions aiming to prevent chronification of subacute LBP had an insignificant preventive impact on pain measures [25].

### Aims and Objectives

There is a shortage of studies on interventions to prevent the chronicity of subacute back pain [25]. Considering the recent increase in knowledge about psychological risk factors, as well as recent findings in cognitive neuroscience regarding emotional and cognitive background factors in the LBP chronicity process, efforts should be made to discover effective preventive methods. Therefore, we will conduct a study aiming to prevent the chronicity of subacute LBP by altering the emotional response to pain using an interoceptive exposure technique (IET).

The aims of this study are as follows:

1. A pilot study will address the efficacy and feasibility of IET in preventing the chronicity of subacute pain. Participants who are found to have a higher risk of pain chronification due to the presence of a biomarker (functional mPFC-NAc synchronicity) will be randomized to the intervention group (n=7) or control group A (n=7; treatment as usual). To assess how well this biomarker predicts LBP chronification, participants who are not found to have the biomarker will be assigned to control group B (treatment as usual) without a defined number of participants.
2. A full-scale study (intervention group: n=20; control group: n=20) will address the efficacy and feasibility of the IET in the prevention of subacute LBP chronicity with the same parameters as in the pilot study.

## Methods

### Participant Selection and Sampling Strategy

All participant recruitment will take place through the pain clinic at the Orton Orthopaedic Hospital and another private occupational health center. All study participants will be outpatients at the pain clinic and the private occupational health center. They are working-aged Finnish citizens who speak Finnish as their first or second language. A health care professional will diagnose the participants’ back pain and assess them based on the inclusion and exclusion criteria.

The inclusion criteria approximately correspond to those of Baliki et al [15]: pain intensity >40/100 on the numeric rating scale (NRS) [26] and back pain duration <16 weeks. Deviating from the abovementioned criteria, we limit the pain duration time more precisely (ie, to 4-10 weeks) to enable commencement of the IET before exceeding the valid 12-week time limit for pain chronicity. Additionally, it will be implied that participants experience at least occasional back pain when lying on their backs. Exclusion criteria resemble those of Baliki et al [15]: other chronic painful conditions, systemic disease, history of head injury, psychiatric diseases, or more than mild depression (score >19), as defined by Beck Depression Inventory-II (BDI-II) [27]. As an addition, exclusion criteria also include self-reported earlier diagnosed prolonged LBP episodes and longer sick leaves due to LBP.

A health care professional (physician or physiotherapist) will ask patients with subacute back pain if they are willing to participate in the study. When a patient is willing to participate, the research secretary will contact the patient and check that he or she meets the research criteria. Then, the health care professional will provide the patient with information about the study procedure and deliver the information and a consent form to the patient, who will sign the consent document. The patient will be told that he or she has the right to stop his or her participation at any time without having to explain the reason. The participant, accompanied by the research secretary, will undergo an fMRI scan and fill out the questionnaires. The feedback from the lumbar magnetic resonance imaging (MRI) will be given to the participants by the clinician. If necessary, proper further examinations and treatments will be provided, and the participants will be excluded from the study.

### Neuroimaging and Data Acquisition

All participants will undergo an fMRI measurement at the Advanced Magnetic Imaging Centre, Aalto University, Espoo, Finland, to determine if they meet the criteria for the biomarker (mPFC-NAc synchronicity), according to the study by Baliki et al [28]. The fMRI is done only in the initial stage, and no post-IET measurement is done to demonstrate possible effects on the biomarker.

The fMRI data will be collected with a 3T Magnetom Skyra whole-body scanner (Siemens Healthineers) and a standard 32-channel head coil. Anatomical T1 magnetization prepared–rapid gradient echo images will be acquired with the following parameters: orientation=sagittal; repetition time (TR)=2530 ms, time to echo (TE)=33 ms; 176 slices, slice thickness=1 mm, gap=50%; phase encoding direction=A-P; field of view=256 mm; and flip angle=7°.

Next, fMRI images will be acquired with a T2* weighted echo planar imaging sequence with the following parameters: orientation=transversal; TR=1570 ms, TE = 30 ms; 38 interleaved slices with no gap, slice thickness=3 mm; phase encoding direction=R-L; field of view=220 mm; flip angle=70°; base resolution=74; and 400 time points/volumes. A total of 4 dummy scans will be acquired for signal stabilization.

We will collect 1 anatomic MRI and 1 fMRI series for each participant. Throughout the fMRI acquisition, the participants will be asked to rate their LBP on a sliding cursor from “no pain” to “worst imaginable pain” (0-10). Participants will be given the following instructions (in Finnish): “Continuously rate the intensity of your low back pain. Indicate the intensity of your low back pain and changes in it by adjusting the cursor to the location corresponding to the intensity of your pain.” Participants will give their answer with a handheld 2-button device. Participants’ answers will not be recorded.

### Functional Connectivity Analysis and Participant Grouping

The functional mPFC-NAc connectivity of each participant will be analyzed with data processing assistant for resting-state fMRI [29]. Slice timing correction will be performed using the first acquired slice (slice number 2) as the reference slice. Images will be realigned, and normalization will be completed using a new segment and the diffeomorphic anatomical registration through exponentiated lie (DARTEL) algebra algorithm [30]. Images will be smoothed by DARTEL using a Gaussian kernel of full width at a half-maximum of 5 mm. Nuisance covariate regression will be performed with the Friston 24-parameter model. White matter signals and cerebrospinal fluid signals will be used as covariates. A 0.01 to 0.1 Hz filter will be applied. The functional region of interests, defined according to the criteria of Baliki et al [28], are spheres with a 10-mm radius. Talairach seed coordinates for the mPFC (x=2, y=52, z=–2) and right NAc (x=10, y=12, z=–8) will be transformed into Montreal Neurological Institute coordinates before calculating mPFC-NAc synchronicity. Participants with z(r) ≥0.25 will be classified as having an increased risk for LBP chronification.

Participants who are found to have a higher risk of pain chronification due to the presence of a biomarker (mPFC-NAc synchronicity) will be randomized to the intervention group (IET) or control group A (treatment as usual). To assess how well this biomarker predicts the chronification of LBP, participants who are not found to have the biomarker will be assigned to control group B (treatment as usual).

Participants who fit the inclusion criteria (based on fMRI) will undergo a lumbar spine MRI examination (1.5 Tesla) to rule out specific causes of the low back symptoms (ie, tumors, evidence of ankylosing spondylitis) or other conditions requiring special attention. Furthermore, the lumbar MRI ensures the homogeneity of the study population. The feedback from the MRI examination will be given to the participants by the clinician briefly in the style of “no specific causes or special factors to be taken into account,” after which the participants will participate according to the protocol. If necessary, proper further examinations and treatments will be provided, and the participants will be excluded from the study.

### Study Procedure

**Figure 1:**
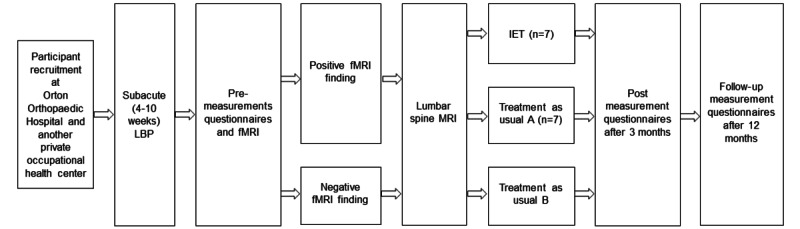
The study procedure. fMRI: functional magnetic resonance imaging; IET: interoceptive exposure technique; LBP: low back pain; MRI: magnetic resonance imaging.

The study procedure is outlined in Figure 1. All participants will complete the study questionnaires before the fMRI, 3 months after completing the IET, and 12 months after the fMRI. Questionnaires will be presented in paper form. Post-IET questionnaires will be sent to participants by paper mail. Questionnaires will be pseudonymized.

Participants at risk for LBP chronification will be classified and categorized within 48 hours after fMRI. High-risk participants will be further pseudo-randomized into the intervention (IET) or control group (A), whereas low-risk participants will form the treatment-as-usual group (B). Participants will be provided with group-specific instructions on the remaining study procedures by email and telephone. Participants in the intervention group will begin practicing the IET within a week after neuroimaging.

### Study Setting

The IET to be used in this study is a modified version of the IET used by Flink et al [31]. The original Swedish version used in the original study was translated into Finnish with small adjustments to the IET without departing from the structure and scope of the original exercise. At the beginning of the study procedure, the participants will be given brief information about the IET. The participants will access the IET through a web-based platform secured by a personal password and a username. The platform can be accessed on a mobile phone or a home computer with internet access. The participants will then be told to perform the 8 minutes and 26 seconds–long IET exercise 3 times a week (Monday, Wednesday, and Friday) in as serene a setting as possible. During the IET, the participants will be asked to relax and calmly focus their attention on the sensation of pain. They will be told to focus on the pain with an open and nonjudgmental attitude and to feel the pain as much as possible without trying to change it or block it. If their attention wanders from the pain, they will be asked to return their focus to the pain sensation. The web-based exercise platform will keep track of the participant’s activity. Platform visits and the time spent committing to the IET will be recorded. In this way, the research team can contact the participant if he or she is not logging on to the platform or if the IET is not carried out completely. The participant will be reached by phone to investigate the problem with attendance. If the participant misses 3 consecutive training sessions (ie, 1 week’s training), he or she will be excluded from the study. The IET intervention is designed to take 12 weeks. The IET exercise (in Finnish) is presented in Multimedia Appendix 1.

### Outcome Assessment and Measurements

The questionnaires used in the study will address conditions of pain experience and psychological distress. All measures are standardized and commonly used in pain research.

For all visits, participants will complete the short form of the Finnish version of the McGill Pain Questionnaire (MPQ), abbreviated as the FPQ [32]. The main component of the MPQ consists of 12 sensory and 4 affective descriptors, which are used to compute the sensory and affective scores, respectively. Radiculopathy scores are quantified from pain locations, which patients shade in with pencil on the MPQ form 15. Depression scores are assessed using the BDI-II [27]. All questionnaires will be given on the same day before brain scanning. The questionnaires are presented in Textbox 1.

**Behavioral measures and corresponding questionnaires to be used in the study, with abbreviations in brackets.**

**Measures and corresponding questionnaires**

- Pain intensity: Numeric Rating Scale (NRS) [26]
- Pain experience: the Finnish Version of the McGill Pain Questionnaire (FPQ) [32]
- Pain-related anxiety: Pain Anxiety Symptoms Scale (PASS-20) [33]
- Anxiety symptoms: State-Trait Anxiety Inventory (STAI) [34]
- Fear of movement: Tampa Scale of Kinesiophobia (TSK) [35]
- Depression: Beck Depression Inventory-II (BDI-II) [27]

Pain intensity will be estimated with the numeric rating scale (NRS), with a pain intensity ranging from “no pain” to “worst pain” (1-10) according to the conventional use of the NRS [26]. The subjective unpleasantness will be measured analogously on the NRS, ranging from “not unpleasant” to “extremely unpleasant.” The primary outcome will be pain intensity on the NRS. Secondary outcomes will be pain experience [32], pain-related anxiety [33], state and trait anxiety [34], fear of movement [35], and depressive symptoms [27] (Textbox 1).

### Data Collection and Statistical Analysis

Data will be obtained before the fMRI, 3 months after completing the IET, and 12 months after the fMRI. Data will be compared between the groups (IET vs treatment as usual in control groups A and B). The data will be analyzed using a repeated-measures analysis (mixed models).

### Ethics Approval

This study has obtained ethical approval from the ethical committee at Helsinki and Uusimaa Health District (HUS/2435/2017). Good research ethics practices will be maintained in the study according to the Declaration of Helsinki.

There will be no inclusion of vulnerable groups (ie, children, prisoners, individuals with mental disorders). No participant reimbursement will be provided to prevent economic factors from impacting the recruitment process. No specific physical health risks can be seen to be involved in participating in the study. Participants will be physically checked with a handheld metal detector before entering the scanner. However, the procedure could cause slight stress for participants and might even provoke pain. The fMRI and MRI procedures could cause some distress for some participants. The IET training might test participants’ motivation.

Anonymity and confidentiality will be ensured by using numerical codes for the participants. Only the research group members will know the participants’ names. Data protection and storage security will be ensured by storing the participant information and questionnaires in a locked cabinet at Orton. Data will be stored behind electronic passwords securely on Orton’s server.

For the duration of the fMRI measurements, all participants will have patient insurance against possible injuries.

## Results

This research project will consist of a pilot randomized controlled trial in which 2 novel methods will be combined: a biomarker as the inclusion criterion and an IET as the intervention. The comparative study design enables evaluation of the efficacy of the IET as well as replication of the biomarker’s validity as a possible clinical screening method. This study will yield valuable new information about both the neural processes driving pain to chronicity and a specific and promising intervention to modulate that process.

As of September 2023, we have not enrolled any participants.

## Discussion

### Principal Findings

The principal finding will be pain intensity. Secondary findings will be pain experience, pain-related anxiety, state and trait anxiety, fear of movement, and depressive symptoms.

### Strengths and Limitations

This study will combine neuroscience and psychology in a novel and innovative way. The study’s strengths will be precise diagnostic criteria, objective selection criteria with measurement methods, and behavioral metrics that have been found to be reliable. Furthermore, the comparative design with 3 groups can be considered a strength.

The small number of participants in the pilot phase can be considered a weakness, as can the completion of the IET at home without objective supervision, which could become a possible source of error.

### Study Significance and Feasibility

The first step of this project was to write a systematic review. It was registered with PROSPERO (Prospective Register of Systematic Reviews; CRD42019053580) and published in 2022 [25]. This study has also been registered at ClinicalTrials.gov (NCT05450263). We have (adjusted to our research design) followed the SPIRIT (Standard Protocol Items: Recommendations for Interventional Trials)-2013 recommendations for clinical trial protocols and related documents [36].

This study will yield valuable new information about both the neural processes driving pain to chronicity and a specific and promising intervention to modulate that process. In addition, this study will offer new insight into the use of mindfulness meditation in pain management. If found to be useful, the IET would offer a cost-effective and reasonable way to develop treatment for back pain.

The potential benefits are improved pain management and quality of life for the individual patient, with the addition of a potentially useful functional pain management method at the societal level.

## Appendix

Multimedia Appendix 1 The interoceptive exposure training (IET) in Finnish.

## Abbreviations

BDI-II: Beck Depression Inventory-II
DARTEL: diffeomorphic anatomical registration through exponentiated lie
fMRI: functional magnetic resonance imaging
FPQ: Finnish version of the McGill Pain Questionnaire
HUS: Helsinki and Uusimaa Health District
IET: interoceptive exposure technique
LBP: low back pain
mPFC: medial prefrontal cortex
MPQ: McGill Pain Questionnaire
NAc: nucleus accumbens
NRS: numeric rating scale
PROSPERO: Prospective Register of Systematic Reviews
SPIRIT: Standard Protocol Items: Recommendations for Interventional Trials
TE: time to echo
TR: repetition time

## Data Availability

The data sets generated during and/or analyzed during this study are available from the corresponding author on reasonable request.
