# Supplementary material for: The Efficacy and Feasibility of an Interoceptive Exposure Technique for Preventing the Transition From Subacute to Chronic Back Pain by Altering the Emotional Response to Pain: Protocol for a Pilot Randomized Controlled Trial
Source: JMIR Res Protoc. 2023 Oct 19;12:e45701. doi: 10.2196/45701 (PMC10623225; doi:10.2196/45701)
Supplement: Multimedia Appendix 1 [file resprot_v12i1e45701_app1.pdf]

Multimedia appendix 1. The interoceptive exposure training (IET) in Finnish (below in English).

Aloita harjoitus keskittymällä ensin hengitykseesi.

Ota rauhallisesti syvään henkeä ja hengitä pehmeästi ulos.

Vedä henkeä sisään rauhallisesti, ja hengitä pehmeästi ulos.

Jokaisella uloshengityksellä anna kehosi rentoutua, niin paljon kuin sinulle on mahdollista juuri nyt.

Hengitä rauhallisesti sisään. Ja hengitä pehmeästi ulos.

Hengityksesi rauhallisesta rytmistä muodostuu tukikohta, josta käsin lähdemme hetken kuluttua tutkimaan kipukokemusta.

Hengityksesi kulkee vapaasti ja rauhallisesti.

Hengityksesi kulkee luonnollisena.

Jos tunnet kipua, anna kivun olla siellä.

Älä yritä etsiä asentoa, jossa kipu tuntuisi lievemmältä.

Jos joudut liikkumaan, tee se hitaasti ja tietoisesti. Tee siitä osa harjoitusta.

Kehosi rentoutuu samalla kun hengität sisään ja ulos, rauhallisesti ja levollisesti.

Kehosi ja mielesi voi rentoutua tässä ja nyt, siten kun sinulle on parasta ja mahdollista.

Hengitä sisään ja hengitä ulos. Syvään ja rauhallisesti.

Jokaisella uloshengityksellä tunnet kehosi rentoutuneemmaksi ja rentoutuneemmaksi.

Jokaisella uloshengityksellä päästä irti tarpeestasi kontrolloida.

Muuntaudu kontrolloijasta tarkkailijaksi.

Ohjaa nyt huomiosi päättäväisesti kipuun.

Jos tunnet kipua monessa osaa kipuasi, valitse jokin kohta. Koe kipu yrittämättä sulkea sitä pois mielestäsi.

Koe kipu yrittämättä muuttaa sitä miksikään.

Kehosi ja mielesi pysyvät mahdollisimman rentona, samalla kun rauhallisesti suuntaat huomiosi ja tarkkaavaisuutesi kipuun.

Jos ajatuksesi alkavat harhailemaan tai keskittymisesi herpaantuu, huomioi se ja palaa rauhallisesti takaisin kivun tuntemukseen.

Tiedosta missä huomiosi on. Tarkkaile tuntemaasi kipua. Samalla kun tarkkailet kipua, pidä mielesi mahdollisimman rentona. Älä yritä ponnistella, vaan pysy tarkkailijan roolissa.

Tietoisuutesi näin seuratessa kiputuntemusta, havaitse mitä tunnet tässä ja nyt. Kipu on pelkkä tuntemus. Se ei uhkaa sinua fyysisesti.

Tarkkaile kipua, miltä se tällä hetkellä näyttää.

Lähesty sitä, vaikka se tuntuisikin herättävän sinussa tuskaa.

Älä tuomitse kipua, älä anna sille mitään merkitystä, vaan jätä se täysin omaan arvoonsa.

Älä arvioi sitä, ainoastaan tarkkaile.

Jos kipu tuntuu yltyvän, jatka siitä huolimatta keskittymistäsi kipuun.

Anna samalla hengityksesi kulkea luonnollisena ja pehmeänä.

Pidä itsesi mahdollisimman rentona.

Huomioi kipu tai särky, tai miksi sitä kutsutaan. Pane merkille miltä se tuntuu. Pidä huomiosi ja tarkkaavaisuutesi kivussa, samalla kun pysyt mahdollisimman rentona.

Tällä hetkellä, hyväksy kivun olemassaolo.

Kysy itseltäsi, mitä sinä havaitset, kun näin keskität tietoisuutesi kipuun. Mikä on kokemuksesi kivusta juuri nyt? Älä arvioi kipua. Älä pohdi muuttuiko se harjoituksen aikana. Pohdi ainoastaan, miten koet kipusi juuri nyt.

Muistathan, että mielelläsi ja kokemuksellasi on voima muokata kipukokemusta. Sinä et ole kipusi.

In English

Begin the exercise by first focusing on your breathing.

Calmly take a deep breath and exhale softly.

Inhale calmly, and exhale softly.

With each exhalation, let your body relax, as much as is possible for you right now.

Inhale calmly. And exhale softly.

The calm rhythm of your breathing forms the base from which we will start after a while to explore the experience of pain.

Your breathing flows freely and calmly.

Your breathing is natural.

If you feel pain, let the pain be there.

Don't try to find a position where the pain will be less.

If you must move, do it slowly and deliberately. Make it part of your exercise.

Your body relaxes as you breathe in and out, calmly, and calmly.

Your body and mind can relax here and now when it's best and possible for you.

Breathe in and breathe out. Deeply and calmly.

With each exhalation, you feel your body more relaxed and relaxed.

With each exhalation let go of your need to control.

Transform from a controller to an observer.

Now direct your attention decisively to the pain.

If you feel pain in several parts of your pain, choose a point. Experience the pain without trying to shut it out of your mind.

Experience the pain without trying to change it for anything.

Your body and mind remain as relaxed as possible, while you calmly direct your attention and attention to the pain.

If your thoughts start to wander or your concentration falters, notice it and calmly return to the feeling of pain.

Know where your attention is. Observe the pain you feel. While observing the pain, keep your mind as relaxed as possible. Don't try to make an effort, just stay in the role of an observer.

As your consciousness thus follows the sensation of pain, notice what you feel here and now. Pain is just a feeling. It does not threaten you physically.

Observe the pain as it appears at the moment.

Approach it, even if it seems to cause you more pain.

Do not judge the pain, do not give it any meaning, but leave it completely on its own merit.

Don't judge it, just observe.

If the pain feels like it's getting worse, continue to focus on the pain anyway.

At the same time, let your breath flow naturally and smoothly.

Keep yourself as relaxed as possible.

Notice the pain or ache, or whatever it's called. Notice how it feels. Keep your focus and attention on the pain, while remaining as relaxed as possible.

In this moment, accept the existence of pain.

Ask yourself what you notice when you focus your awareness on the pain like this. What is your experience with pain right now? Don't judge the pain. Don't wonder if it changed during the practice. Just think about how you feel your pain right now.

Remember that your mind and experience have the power to shape the pain experience. You are not your pain.
